# Comorbidity burden and health-related quality of life in men with advanced prostate cancer

**DOI:** 10.21203/rs.3.rs-2572781/v1

**Published:** 2023-02-16

**Authors:** Roberto Benzo, Patricia I. Moreno, Rina S. Fox, Carlos A. Silvera, Emily A. Walsh, Betina Yanez, Raymond R. Balise, Laura B. Oswald, Frank J. Penedo

**Affiliations:** Department of Psychology, University of Miami; Department of Public Health Sciences, University of Miami Miller School of Medicine; University of Arizona Cancer Center; University of Miami Miller School of Medicine; Department of Psychology, University of Miami; Department of Medical Social Sciences, Northwestern University Feinberg School of Medicine; Department of Public Health Sciences, University of Miami Miller School of Medicine; Department of Health Outcomes and Behavior, Moffit Cancer Center; Department of Psychology, University of Miami

**Keywords:** Cancer, Comorbidity, Oncology, Patient Reported Outcome Measures, Prostatic Neoplasms, Quality of Life

## Abstract

**Purpose:**

Identifying clinically relevant comorbidities and their effect on health-related quality of life (HRQoL) outcomes among men with advanced prostate cancer (APC) can inform patient care and improve outcomes; however, this is poorly understood. The aim of this observational study was to examine the prevalence of comorbidities, and the relationship of comorbidity burden to HRQoL and other patient-reported outcomes (PROs) among men with APC.

**Methods:**

Participants were 192 men (average age 68.8) with APC (stage III or IV) who completed a psychosocial battery including measures of sociodemographic factors, HRQoL and other PROs, and the Charlson Comorbidity Index (CCI). Hierarchical multiple regression analysis was used to examine the relationships between CCI, HRQOL, and PROs.

**Results:**

The vast majority (82%) of participants had at least one comorbidity, with the most common being: hypertension (59%), connective tissue disease or arthritis (31%), diabetes (24%), and problems with kidneys, vision, or another organ (24%). After controlling for covariates, regressions showed that a higher CCI score was significantly associated with worse HRQoL (*p* < 0.001), lower levels of positive affect (*p* < 0.05), and higher levels of depression (*p* < 0.05), fatigue (*p* < 0.001), pain (p < 0.01), stress (*p* < 0.01), and cancer-specific distress (*p* < 0.05).

**Conclusions:**

Comorbidities were common among men with APC, and a greater CCI score was associated with detriments in several domains of HRQoL and other PROs. Our findings show the need to address comorbidities in the presence of a cancer diagnosis and subsequent treatment.

## 1. Introduction

Prostate cancer (PC) is the most commonly diagnosed cancer and the second leading cause of cancer-related death among men in the United States (US) [1]. Men with advanced prostate cancer (APC) (i.e., stage III or IV) have a worse prognosis than men with localized PC; for example, the National Cancer Institute - Surveillance, Epidemiology, and End Results database estimates > 99% five-year survival rates for men diagnosed with local and regional PC, and 31% when diagnosed with metastatic [2]. While treatment can increase life expectancy for patients with APC, survival benefits may be offset by chronic and debilitating disease- and treatment-related side effects that can compromise their health-related quality of life (HRQoL) [3] [4]. For example, androgen deprivation therapy (ADT) is the standard of care hormonal therapy for men with APC [5], which has been associated with vasomotor flushing, anemia, bone density loss, fatigue, osteoporosis, fractures, obesity, insulin resistance, diabetes, and cardiovascular disease (CVD) [4].

The average age of PC diagnosis is 66 years [6]. As age increases, the risks of developing and dying from PC increase [7, 8], as does the prevalence of comorbid conditions [9]. For example, in 2012, about 26% of the United States population had multiple chronic conditions; and in adults older than 65 years, the prevalence of multiple chronic conditions was as high as 81% [10]. Among older adults with cancer, more than half have at least one comorbidity [11], and a greater comorbidity burden in PC has been associated with worse all-cause and PC-related survival [3, 12–14]. Some comorbidities appear to be more strongly related to health outcomes in PC than others. For example, in a sample of 1,031 veterans with PC, Chamie et al. [12] found that moderate to severe chronic obstructive pulmonary disease (COPD) had the strongest association with risk for non-PC mortality (HR 5.46); followed by diabetes with end-organ damage (HR 4.27), needing a mobility device (HR 3.29), peripheral vascular disease (HR 2.77), and diabetes without end-organ damage (HR 2.32). Other work has found that comorbidity burden, as measured by the Charlson Comorbidity Index (CCI), is a strong predictor of mortality [13], suggesting that comorbidities should be considered when making PC treatment choices. Unfortunately, the lack of a standardized comorbidity assessment tool has limited the consideration of comorbidity burden during clinical decision-making in PC [15].

Previous research evaluating comorbidity burden in PC has typically examined how specific chronic conditions impact treatment options and cancer-specific and non-cancer-specific mortality [3, 12, 13]. However, the impact of the overall comorbidity burden on domains of HRQoL and patient-reported outcomes (PROs) in APC survivors remains poorly understood [16]. Additionally, little to no work has evaluated how these relationships may differ by race, which is important given the well-established racial disparities in PC survival [17] and in prevalence of chronic diseases [18]. Thus, the aims of this study are to 1) report the prevalence of comorbidities among men with APC and 2) to examine the relationship of comorbidity burden to HRQoL and PROs.

## 2. Methods

This study included baseline data from APC participants enrolled in a behavioral randomized clinical trial designed to improve HRQoL and reduce symptom burden. Results from the trial have been previously published [19–21].

### 2.1 Participants

Participants were recruited from Northwestern Memorial Hospital and the Robert H. Lurie Comprehensive Cancer Center of Northwestern University, the Jesse Brown Veterans Affairs Medical Center, Rush University Medical Center, and two Northwestern Medicine locations in Lake County, Illinois. The study CONSORT diagram, details about recruitment, and descriptions of the original study conditions have been previously published [19]. Eligible men were ≥ 50 years old, fluent in English at the 6th-grade level or higher, diagnosed with APC (stage-III vs. IV), and had been treated with ADT within the 12 months before enrollment. Men were excluded if they had been diagnosed with another primary cancer in the previous five years other than non-melanoma skin cancer, underwent inpatient psychiatric treatment for mental illness in the past six months, reported active substance or alcohol dependency, were diagnosed with an acute or chronic immune system condition or received a score < 20 on the Mini-Mental State Examination [22].

### 2.2 Procedures

Institutional Review Board approval was received before enrollment, and the protocol is available in more detail on ClinicalTrials.gov (NCT03149185) [19]. All participants provided informed consent and were enrolled between January 2013 and November 2016. At baseline, six months, and 12 months post-baseline, participants attended in-person appointments where they completed a battery of psychosocial assessments, and clinical information was obtained from participants’ medical records. Data used in this study are limited to select measures collected at baseline so that the effects of the behavioral intervention did not confound associations between comorbidity burden and PROs.

### 2.3 Measures

#### 2.3.1 Sociodemographic and medical information.

We collected age, body mass index (BMI), PC stage (III or IV), and years since diagnosis via medical chart review. Income (≥ $35,000) and race were collected via self-report.

#### 2.3.2 Comorbidity Burden.

Comorbidities were self-reported and verified via medical chart review. We calculated a comorbidity score using a previously adapted version of the CCI [20, 21], which accounts for 19 diseases that were weighted based on their association with mortality. Although hypertension was not originally included in the CCI, it is included in our adapted version and assigned one point. Hypertension was added because it is the most important risk factor for CVD, which is the leading cause of death in PC and has been linked to ADT [23]. We used the weighting scheme from the original CCI, where a higher score indicates a higher comorbidity burden [24].

#### 2.3.3 Health-Related Quality of Life and PROs

##### Health-Related Quality of Life (HRQoL).

HRQoL was measured using the 27-item Functional Assessment of Cancer Therapy- General (FACT-G) scale [25]. Participants rated the extent to which each item applied to them in the previous seven days using a five-point scale ranging from 0-”not at all” to 4-”very much,” where a higher score indicates better quality of life. The FACT-G evaluates four dimensions of well-being (physical, social, emotional, and functional), which can be summed to yield a total score ranging from 0 to 108. Only the total score was used in our analyses. The FACT-G is a psychometrically strong measure commonly used in oncologic samples.

##### Depression.

Depression was measured using the PROMIS-Depression Item Bank computer adaptive test [26]. PROMIS assessments are T-scored, where a mean score of 50 and a standard deviation of 10 represent the average U.S. population score. Each item is rated on a five-point scale ranging from 0- “Never” to 4-”Always,” and a higher score indicates greater depressive symptomatology. The measure has been well-validated for use in cancer samples [27].

##### Fatigue.

Fatigue was measured using the Fatigue Symptom Inventory (FSI) [28], a 14-item self-report measure on which a higher score indicates greater fatigue. The perceived interference score was used in the current analyses. It was calculated by averaging the seven items, which assess the degree to which fatigue has interfered with daily life in the past seven days. Each item was rated on an 11-point scale from 0-”Not at all fatigued” to 10-”As fatigued as could be.” A global score can be obtained for items 1–13, ranging from 0 to 130; lower points on the scale denote less acute fatigue-related problems.

##### Pain.

Pain was measured using the 15-item McGill Pain Questionnaire short form [29], where 11 items measure the sensory component, and the remaining four assess the affective component. Each item is rated on a four-point scale ranging from 0-”none” to 3-”severe,” with a score ranging from 0 to 45, and a higher score indicates more pain. This instrument has strong psychometric properties [30].

##### Perceived Stress.

Perceived stress was measured using the 14-item Perceived Stress Scale (PSS) [31]. Participants rated items assessing the frequency of thoughts and feelings related to their lives over the past month on a five-point Likert scale ranging from 0-”Never” to 4-”Very often.” Positively worded items are reverse-coded before scoring, and a single-sum score is calculated. The total scores range from 0 to 56, where a higher score indicates more stress.

##### Cancer-Specific Distress.

We measured cancer-specific distress using the 22-item Impact of Events Scale-Revised (IES-R) [32]. Participants rated the level of cancer-specific distress caused by intrusive thoughts, avoidance, and hyperarousal over the past week on a five-point scale ranging from 0-”not at all” to 4- “extremely.” The three sub-scales yield a total score ranging from 0 to 88, with higher scores indicating greater cancer-specific distress. The measure has acceptable psychometric properties in cancer samples [33].

##### Positive Affect.

We measured positive affect using the 20-item modified version of the Affect Balance Scale (ABS) [34]. Participants rated the frequency with which they experienced various emotions during the past week on a five-point scale ranging from 0-”never” to 4-”always.” Items assessing negative affect were reverse coded, and then a total score was calculated, with higher scores indicating greater positive affect.

### 2.4 Data Analysis

Descriptive statistics were calculated for sociodemographic factors, medical factors, and HRQoL and PROs. We assessed all variables for normality via visual inspection and checked for skewness (less than +/− 2) [35, 36]. Numeric data were reported as means and standard deviations (SD). The internal consistency for each of the HRQoL measures are reported the primary outcome publication [19].

We computed the prevalence of each comorbidity and the number of comorbid conditions (0, 1, 2, or ≥ 3) for the overall sample and by race (White vs. Minorities). The Minorities group (40% of the total sample, n = 77), consisted of 90% Black (n = 69), 6% multiracial (n = 5), and 4% Asian (n = 3). We tested for significant differences between White vs. Minorities using the chi-square test for categorical variables.

We used hierarchical multiple regression analysis to examine the relationship between the CCI score and various HRQoL domains and PROs, while controlling for covariates [37]. We used the change in R^2^ (ΔR^2^) to assess the incremental variance accounted for by the CCI score after controlling for sociodemographic and medical factors. We report the unstandardized coefficients (*b*), standard error (*SE*), and p-value for each significant finding. Our models were evaluated in three steps: (1) sociodemographic variables, (2) medical variables, and (3) CCI score. The first model (Model 1) included the sociodemographic variables, which include: age, BMI, income (1-” $35,000”; 0-”<$35,000”), and race (1-”White”; 0-”Minorities”). The second model (Model 2) included the≥ medical variables, which included: stage IV PC (1-”yes”; 0-”no”), ADT in the past six months (1-”yes”; 0-”no”), radiation in the past six months (1-”yes”; 0-”no”), chemotherapy in the past six months (1-”yes”; 0-”no”), radical prostatectomy (RP; 1-”yes”; 0-”no”), and years since diagnosis. The third model (Model 3) included the CCI score. Age, years since diagnosis, and BMI were grand-mean-centered [24]. An alpha level ≤ 0.05 was considered significant for all analyses. R-studio (Version 1.4.1106) software program was used to analyze the data.

## 3. Results

Sociodemographic factors, medical factors, HRQoL, and other PROs for the 192 participants are summarized in Table 1. The mean age of participants was 68.8±8.9 years and the average BMI (28.8 kg/m^2^) was in the overweight range. Most participants had a family income of less than $35,000 (65%) and were White (59%). Less than half had metastatic cancer (42%), and the average time since cancer diagnosis was almost five years.

### 3.1 Prevalence of Comorbidities

The counts and percentages of comorbidities are presented in Table 2. Participants with no comorbidities comprised 18% of the sample, 27% had one, 25% had two, and 30% had three or more comorbidities. Most participants had hypertension (59%), and approximately one-third reported having “connective tissue disease or arthritis” (31%). About one quarter (24%) of the sample reported having diabetes, and one quarter (23%) reported problems with their “kidneys, vision, or another organ.” Lastly, 10% of participants reported having stomach ulcers, and 10% reported having a lung-related illness. No significant differences were observed in the number of comorbidities between Whites and the Minorities in our sample. However, participants in the Minorities group had a significantly higher prevalence of diabetes compared to Whites (32% vs. 23%, respectively; *p* = 0.024). We did not observe any other differences in comorbidity prevalence by race.

Results of the hierarchical multiple regression analyses results can be found in Tables 3 and 4. After controlling for sociodemographic and medical factors (Model 3), a higher CCI score was associated with worse HRQoL (*b*=−2.0, *SE* = 0.56, *p* < 0.001), lower positive affect (*b*=−1.01, *SE* = 0.45, *p* = 0.02), and higher levels of depression (*b* = 0.73, *SE* = 0.34, *p* = 0.03), fatigue (*b* = 1.94 *SE* = 0.53, *p* < 0.001), pain (*b* = 1.03, *SE* = 0.31, *p* = 0.001), stress (*b* = 0.77, *SE* = 0.29, *p* = 0.010), and cancer-specific distress (*b* = 1.05, *SE* = 0.52, *p* = 0.004). The changes in R^2^ between the first and second models were not statistically significant across outcomes. However, we did observe significant changes in *R*^2^ in the third model when examining HRQoL (*ΔR*^*2*^ = 8%, *F* = 12.71, *p* < 0.001), depression (*ΔR*^*2*^ = 3%, *F* = 4.57, *p* = 0.034), fatigue (*ΔR*^*2*^ = 8%, *F* = 13.50, *p* < 0.001), pain (*ΔR*^*2*^ = 7%, *F* = 11.09, *p* = 0.001), stress (*ΔR*^*2*^ = 5%, *F* = 6.92, *p* = 0.009), cancer-specific distress (*ΔR*^*2*^ = 3%, *F* = 4.03, *p* = 0.047), and positive affect (*ΔR*^*2*^ = 3%, *F* = 4.98, *p* = 0.027).

## 4. Discussion

This work extends the literature by examining the prevalence of comorbidity burden and its relationship to several domains of HRQoL and PROs in men with APC. Our main finding was that a higher CCI score was associated with lower levels of HRQoL and positive affect; and higher levels of depression, fatigue, pain, stress, and cancer-specific distress. Also, 82% of participants had at least one comorbidity, with hypertension being the most common (59%), followed by connective tissue disease or arthritis (31%), diabetes (24%), and problems with kidneys, vision, or another organ (24%).

Consistent with our findings, Xiao et al. found similar prevalence rates of comorbidities in men with PC using registry and statewide databases in Florida [38]. Specifically, 42% of patients had hypertension (vs. 59% in our sample), 11% had diabetes, and 19% had “endocrine disorders, nutritional/metabolic, or immunity” (vs. 24% reporting diabetes in our sample), 24% had genitourinary system disease (vs. 24% reporting problems with kidneys, vision, or another organ in our sample), and 9% had chronic pulmonary disease (vs. 10% reporting lung illnesses in our sample) [38]. In contrast, the authors reported a prevalence of musculoskeletal and connective tissue disease that was lower (12%) than our sample (31% connective tissue disease or arthritis). Chaime et al. also reported comorbidity prevalence in men with PC; however, they did not include patients that had more than a single comorbid condition [12]. In contrast to our findings, authors reported much lower rates of comorbidities, with only 13% having diabetes and 1% having connective tissue disease. Discrepancies between our results may be due to geographical differences within the US and because Chaime et al. only included patients with no more than one comorbid condition.

Our findings related to the overall prevalence of comorbidities are aligned with previous studies [38, 39]. Chambers et al. examined the comorbidity burden among 1,064 Australian men diagnosed with PC from 10 public hospitals in Queensland [39]. Consistent with our findings, authors reported that 83% of PC patients had at least one comorbidity (vs. 82% in our sample), where 53% had one to two comorbidities (vs. 53%), and 31% had three or more comorbidities (vs. 30%) [39]. Our findings of comorbidity prevalence are important because comorbidity burden is associated with a higher risk for non-PC mortality in PC survivors and thus should be accounted for when developing a treatment plan [12–14].

When comparing comorbid conditions by race, Xiao et al. reported that White participants had on average 2.25 comorbidities (vs. 2.0 in Whites in our sample), 22% had one (vs. 22%), 18% had two (vs. 30%), and 13% had three comorbidities (vs. 27%) [38]. Black participants in their sample had, on average, 2.5 comorbidities (vs. 1.9 in minorities in our sample), 21% had one (vs. 31%), 17% had two (vs. 22%), and 13% had three comorbidities (vs. 31%). In contrast to our findings, authors reported that Blacks had a significantly higher mean number of comorbidity conditions than Whites, and a higher proportion of Blacks (77%) had at least one comorbid condition compared to Whites (75%). Differences between our findings could be because Xiao et al. included participants with all stages of PC, where we only included those with APC. Also, our sample was recruited in the state of Illinois, while theirs was recruited in Florida. Lastly, differences could be attributed to the different instruments used to record comorbidities (CCI vs. Elixhauser Comorbidity Index).

Aligned with the literature, our hierarchical multiple regression analyses showed that a higher CCI score was associated with lower levels of HRQoL and positive affect and higher levels of depression, fatigue, pain, stress, and cancer-specific distress, even after controlling for sociodemographic and medical variables. Also, the significant increase in R^2^ due to the addition of the CCI score, suggests that comorbidity burden is negatively associated with HRQoL and PROs, independent of sociodemographic and medical factors. Similar to our study, Reeve et al. also examined the impact of baseline comorbidity on HRQoL outcomes [40]; authors found that the number of comorbidities was associated with poorer physical health (measured by the SF-36) in men receiving brachytherapy. Arredondo et al. examined the impact of comorbidity on general and disease-specific HRQoL in men undergoing RP for PC [41]; authors found that the presence of more comorbidities was associated with worse HRQoL domains, including physical function, role physical, vitality, bodily pain, general health, and physical component summary. These findings suggest that understanding total comorbidity burden, regardless of the condition, provides an insight into the patient’s risk for impairments in HRQoL and PROs.

While it is well established that detriments in physical and psychosocial functioning are associated with specific treatment modalities and pretreatment functioning [42], our findings are important because the comorbidity burden could further exacerbate the effect treatment has on these PROs and overall HRQoL. In addition, nearly a quarter (23%) of PC patients experience treatment regret, and regret is more frequently reported when patients experience unwanted physical, psychosocial, and oncological outcomes [42]. For this reason, greater efforts should be made to educate patients and providers about the possible consequences and effectiveness of treatments, as this may help anticipate detriments in HRQoL and limit the feeling of treatment regret.

While the only difference in comorbidities observed by race in our study was diabetes prevalence (18% vs. 32%, respectively), the literature shows that comorbidity burden is more prevalent among Blacks than Non-Hispanic Whites [9, 43, 44]. Common comorbidities among racial/ethnic minorities with PC include, but are not limited to, hypertension, diabetes, ulcers, liver disease, obesity, depression, urinary issues, and sexual dysfunction [44, 45]. In addition, Black men in the United States have a 1.5 times greater chance of developing PC than White men and are 2.2 times more likely to die from the disease [45]. Thus, additional attention should be placed on the comorbidity burden among minorities.

### 4.2 Clinical Implications

Our findings show that comorbidities are common among men with APC, and a greater comorbidity burden is associated with poorer HRQoL and several PROs. This is important because comorbidities are positively associated with an increased risk for non-PC mortality among PC survivors [12–14]. Also, CVD is a leading cause of mortality in PC patients, and ADT may worsen their cardiovascular risk [23]. Thus underscoring the need for medical providers to take into account the impact of comorbidity burden on HRQoL and PROs, particularly when considering treatment options. For example, standard therapies such as ADT could further exacerbate detriments to HRQoL and PROs.

The importance of comorbidity burden in PC is emphasized by the International Society of Geriatric Oncology, which recommends that older men should be managed according to the severity of comorbid conditions they experience and not according to their chronological age [3]. While it is the primary responsibility of the physician to assess patients’ risk due to comorbid conditions, additional interventions could target survivors to increase their knowledge of such risks and provide evidence-based lifestyle recommendations (e.g., stress management, physical activity, diet) to reduce the risk of non-PC mortality. In summary, our findings can inform healthcare providers and researchers working with APC patients to improve patient care and outcomes and highlight gaps in care and the need for interventions to reduce the comorbidity burden in this population.

### 4.3 Limitations

Our study presented several limitations. First, given that our sample was cross-sectional, we cannot account for reverse causality, as the comorbidity could have developed after PC diagnosis and receipt of related treatments. Second, we had a relatively small sample, which may have limited us from detecting significant observations that were present. For example, our sample of minorities was mixed, and given our small sample size, we could not perform sub-analyses to determine the burden for each race included in our sample. Third, our sample was geographically limited, which limit the generalizability of our findings. Our study also has strengths worth noting. First, this is one of the few studies examining the prevalence of comorbidities among men with APC as opposed to localized disease. Second, our study’s comprehensive battery of surveys allowed us to explore the relationships between overall comorbidity burden and various domains of HRQoL and PROs.

### 4.4 Conclusion

In conclusion, our findings suggest that comorbidities are common among men with APC, and greater comorbidities are associated with detriments in several domains of HRQoL and PROs. Further research is warranted to understand the etiology of comorbidities among APC survivors and to inform the design of interventions to prevent the development and exacerbation of comorbidities.

## Figures and Tables

**Table 1:** Sample Characteristics

	Full Sample (N = 192)	Range [min, max]
**Sociodemographic Factors**		
Age, mean (SD)	68.8 (8.9)	51.0, 94.0
BMI (SD)	28.8 (5.1)	17.7, 47.3
Family annual income ≥ $35,000, n (%)	125 (65.1)	N/A
Whites, n (%)	113 (58.9)	N/A
Minorities, n (%)	77 (40.1)	N/A
**Medical Factors**		
PC Stage IV (vs. Stage III), n (%)	81 (42.2)	N/A
ADT 6-months prior to baseline, n (%)	131 (68.2)	N/A
Radiation therapy 6-months prior to baseline, n (%)	39 (20.3)	N/A
Chemotherapy 6-months prior to baseline, n (%)	9 (4.7)	N/A
Prostatectomy 6-months prior to baseline, n (%)	98 (51.0)	N/A
Years since diagnosis, mean (SD)	4.7 (5.3)	0.01, 30.0
**Cancer-Related Health Outcomes**		
Quality of Life Total Score, mean (SD)	84.4 (13.9)	34.0, 108
Depression Score, mean (SD)	46.5 (8.46)	34.2, 76.9
Fatigue Score, mean (SD)	9.03 (12.6)	1.0, 60.0
Pain Score, mean (SD)	5.83 (7.44)	0.0, 37.0
Perceived Stress Score, mean (SD)	16.8 (7.27)	2.0, 37.0
Cancer-Specific Distress Score, mean (SD)	11.9 (12.3)	0.0, 65.0
Positive Affect Score, mean (SD)	79.2 (11.0)	42.0, 99.0

Abbreviations: %, percentage; ADT, androgen deprivation therapy; n, count; PC, prostate cancer; SD, standard deviation. Note: for PC Stage IV, all others had stage III.

**Table 2:** Overall Prevalence of Comorbidities and by Race

Comorbidity	Overall	Whites	Minorities	Chi^2^ p-value
1. Hypertension (n = 186)	109 (58.6%)	59 (53.6%)	60 (65.8%)	0.098
2. Connective tissue disease or arthritis (n = 184)	57 (30.5%)	34 (30.6%)	23 (31.5%)	0.900
3. Diabetes (n = 185)	44 (23.8%)	20 (18.0%)	24 (32.4%)	**0.024** *
4. Problems with kidney, vision, or another organ (n = 184)	43 (23.4%)	29 (26.1%)	14 (19.2%)	0.276
5. Stomach ulcers (n = 184)	19 (10.3%)	10 (9.0%)	9 (12.3%)	0.469
6. Lung illness (n = 185)	19 (10.3%)	13 (11.7%)	6 (8.1%)	0.429
7. Kidney problems (n = 183)	16 (8.7%)	10 (9.0%)	6 (8.2%)	0.838
8. Circulatory problems (n = 183)	13 (7.1%)	8 (7.3%)	5 (6.8%)	0.880
9. Heart attack (n = 185)	13 (7.0%)	8 (7.2%)	5 (6.8%)	0.095
10. Brain stroke (n = 185)	10 (5.4%)	6 (5.4%)	4 (5.4%)	1.000
11. Memory problems (n = 185)	6 (3.2%)	3 (2.7%)	3 (4.1%)	0.611
12. Other cancer except skin, prostate, or invasive bladder cancer (n = 185)	6 (3.2%)	4 (3.6%)	2 (2.7%)	0.735
13. Heart is working < 30% or congestive heart failure (n = 185)	5 (2.7%)	4 (3.6%)	1 (1.4%)	0.355
14. Hepatitis A or fatty liver (n = 184)	4 (2.2%)	4 (3.6%)	0 (0.0%)	0.097
15. Hepatitis B or C or cirrhosis (n = 184)	1 (0.5%)	1 (0.9%)	0 (0.0%)	0.411
16. HIV or AIDS (n = 185)	0 (0.0%)	0 (0.0%)	0 (0.0%)	-
**Comorbidity Severity**				
Average Comorbidities	1.92	1.97	1.88	-
Total Comorbidities Reported	190	77	113	0.521
Total with 0 Comorbidities (%)	34 (17.9%)	16 (20.8%)	18 (15.9%)	
Total with 1 Comorbidity (%)	52 (27.4%)	17 (22.1%)	35 (31.0%)	
Total with 2 Comorbidities (%)	48 (25.3%)	23 (29.9%)	25 (22.1%)	
Total with 3 + Comorbidities (%)	56 (29.5%)	21 (27.3%)	35 (31.0%)	

* *p* < 0.05;

** *p* < 0.01;

*** *p* < 0.001;

All significant relationships (p < 0.05) are bolded.

Abbreviations: n, count who reported “Yes” to the comorbidity category; p-values represent statistical difference between Whites versus Minorities.

**Table 3:** Hierarchical Regressions Analyses of Predictors of Health-Related Quality of Life and PRO – Positive Associations

	HRQoL			Positive Affect		
Independent Variables	Model 1	Model 2	Model 3	Model 1	Model 2	Model 3
**Personal Factors (Step 1)**						
Age	**0.304** *	**0.338** *	**0.403** **	0.146	0.158	0.191
BMI	−0.067	−0.006	0.012	0.012	0.027	0.037
Income	2.652	2.034	−0.115	−0.400	0.441	−0.640
White	0.334	−1.350	−0.193	−2.814	−3.577	−2.995
**Medical Factors (Step 2)**						
PC Stage IV	-	0.970	0.845	-	1.741	1.678
ADT in Past 6-months	-	10.338	10.525	-	**15.150** **	**15.245** **
Radiation	-	−3.933	−4.276	-	0.389	0.216
Chemo	-	4.858	3.940	-	2.387	1.925
RP	-	**6.082** *	4.495	-	0.814	0.015
Years Since Diagnosis	-	−0.132	−0.100	-	−0.016	0.001
**Comorbid Factor (Step 3)**						
CCI	-	-	**1.998** ***	-	-	**−1.005** *
R^2^	0.043	0.096	0.172	0.03203	0.086	0.118
R^2^ Change		0.053	**0.076** ***	-	0.054	**0.032** *

* *p* < 0.05;

** *p* < 0.01;

*** *p* < 0.001;

All significant relationships (p < 0.05) are bolded; Abbreviations: ADT = androgen deprivation therapy in past six months, BMI = body mass index, CCI = Charlson Comorbidity Index, Chemo = chemotherapy in the past six months, HRQoL = health-related quality of life, PROs = Patient reported outcome, RP = radical prostatectomy in the past six months, and PC = prostate cancer

## Data Availability

The data are currently used for secondary analyses and manuscript development. The data that support the study may be available upon request with permission from the researchers who collected the data.
